# Metabolic Features of Ganjang (a Korean Traditional Soy Sauce) Fermentation Revealed by Genome-Centered Metatranscriptomics

**DOI:** 10.1128/mSystems.00441-21

**Published:** 2021-08-03

**Authors:** Byung Hee Chun, Dong Min Han, Hyung Min Kim, Dongbin Park, Da Min Jeong, Hyun Ah Kang, Che Ok Jeon

**Affiliations:** a Department of Life Science, Chung-Ang University, Seoul, Republic of Korea; Pontificia Universidad Catolica de Chile

**Keywords:** metagenome, fermentation, ganjang, genome centered, metabolic pathway, metatranscriptome, soy sauce

## Abstract

The taste and quality of soy sauce, a fermented liquid condiment popular worldwide, is greatly influenced by microbial metabolism during fermentation. To investigate the fermentative features of ganjang (a Korean traditional soy sauce), ganjang batches using meju (fermented soybean) bricks and solar salts were prepared, and organic compounds, microbial communities, metagenomes, and metatranscriptomes of ganjang were quantitively analyzed during fermentation. Polymeric compound analysis in the ganjang treated with/without microbial inhibitors revealed that indigenous enzymes of meju bricks might be primarily responsible for degrading polymeric compounds. Through metagenome binning and microbe sequencing, 17 high-quality genome sequences representing all major ganjang microbiota were obtained, and their transcriptional expressions were quantitatively analyzed by mapping metatranscriptome reads normalized by spike-in RNA sequencing to the 17 genomes, which revealed that microbial metabolism might primarily occur while meju bricks are in the ganjang solution and decrease significantly after the removal of meju bricks. Metabolic pathways for carbohydrates, proteins, and lipids of the major ganjang microbiota were reconstructed, and their metabolic genes were transcriptionally analyzed, revealing that facultative lactic acid fermentation by Tetragenococcus was the major fermentation process active in the ganjang fermentation and that aerobic respiration by facultatively aerobic bacteria such as Chromohalobacter, Halomonas, and Marinobacter was also an important metabolic process during fermentation. Although the abundances of *Fungi* and the corresponding transcriptional expression levels were generally much lower than those of *Bacteria*, our analysis suggests that yeasts such as Debaryomyces and Wickerhamomyces might be in large part responsible for producing biogenic amines and flavors.

**IMPORTANCE** The taste and quality of soy sauce, a popular fermented liquid condiment worldwide, is greatly influenced by microbial metabolism during fermentation. Spontaneous fermentation of ganjang (a Korean traditional soy sauce) in a nonsterile environment leads to the growth of diverse bacteria and fungi during fermentation, making it difficult to understand the mechanism of ganjang fermentation. Genome-centered metatranscriptomic analysis, combined with organic compound analysis, quantitative metagenome and metatranscriptome analyses, and metabolic pathway reconstruction and expressional analysis of the major ganjang microbiota during fermentation, would provide comprehensive insights into the metabolic features of ganjang fermentation.

## INTRODUCTION

Soy sauce, a traditional fermented liquid condiment that originated in East Asia, is popularly used in cooking all over the world (1). It varies in flavor and taste among different countries because of the differences in raw materials and manufacturing processes (2). Generally, in Korea, soy sauce is made using soybeans alone, whereas in Japan and China, it is made using a combination of soybeans and wheat flour (3). However, soy sauce is produced through a two-step fermentation process, i.e., solid-state fermentation (koji in Japan, meju in Korea, and qu in China) and brine fermentation, regardless of its country of origin (1). In traditional soy sauce production, spontaneous fermentation in a nonsterile environment leads to the growth of diverse bacteria and fungi (4–6), making it difficult to understand the fermentation features of soy sauce.

An understanding of the metabolic features of bacteria and fungi during soy sauce fermentation is necessary because the taste and quality of soy sauce are greatly influenced by microbial metabolism. As a basic step toward obtaining an understanding of soy sauce fermentation, microbial communities have been analyzed using culture-dependent and -independent approaches such as the plate method and PCR-based denaturing gradient gel electrophoresis during soy sauce fermentation (7–12). However, these approaches have limitations with respect to enabling an understanding of soy sauce fermentation features because of the lack of information regarding metabolic functions and the behavior of soy sauce microbiota. Metagenomic sequencing using next-generation sequencing was employed to analyze microbial succession and functional potential during soy sauce fermentation (13), but the metabolic genes and activities of soy sauce microbiota were not explored. To the best of our knowledge, none of the studies have investigated the metabolic features of soy sauce microbiota during fermentation.

Metagenome and metatranscriptome analyses have provided useful insights into taxonomic as well as metabolic features of complex microbiota in various environments, including traditional fermented foods (14–19). Ganjang is a Korean traditional soy sauce that is made by soaking and separating meju (fermented soybean) bricks in brine; this preparation is similar to that used for the preparation of other soy sauces. In this study, genome-centered metatranscriptomic analysis, combined with metagenome sequencing, genome sequencing of isolated microbes, metabolic pathway reconstruction, and quantitative metatranscriptomic sequencing, was performed during ganjang fermentation to unravel the taxonomic and metabolic features of ganjang fermentation. Such a strategy will provide a better understanding of ganjang fermentation.

## RESULTS

### General features of ganjang fermentation.

**FIG 1:**
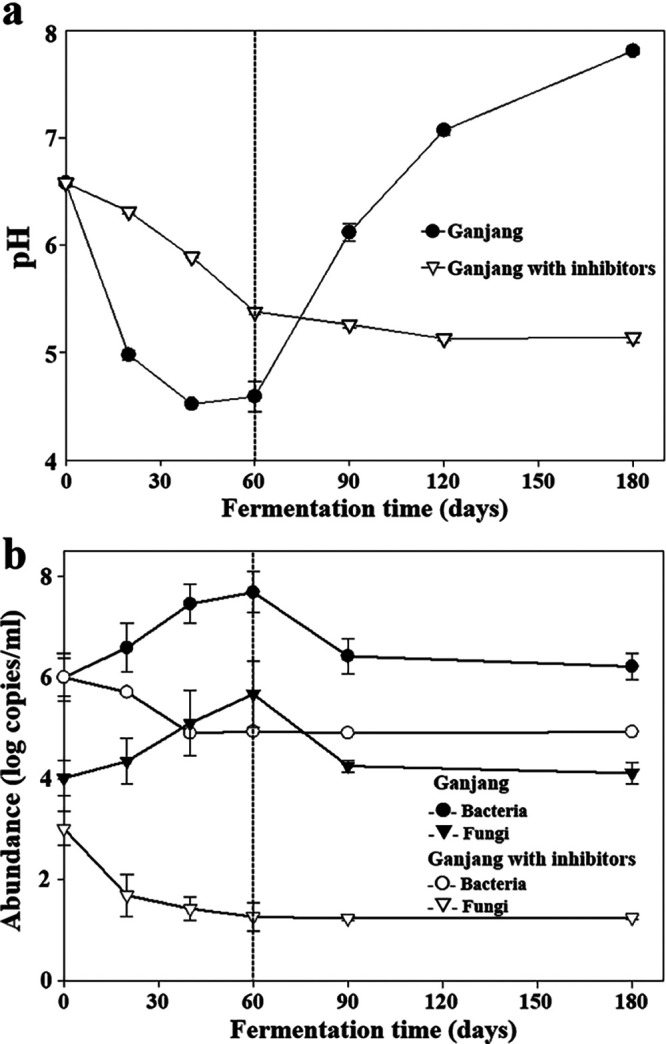
Profiles of pH (a) and bacterial and fungal abundances (b) in ganjang with and without microbial inhibitors during fermentation. The dotted lines indicate the time at which the meju bricks were removed from the ganjang solution. The error bars indicate standard errors; data were measured in triplicate.

Because meju bricks used for the preparation of the ganjang batches contain enzymes produced during meju fermentation, indigenous enzymes in meju may also play important roles, along with microbes, during ganjang fermentation. Therefore, to differentiate the contributions of microbes to ganjang fermentation from those of indigenous enzymes in meju, a ganjang batch treated with bacterial and fungal inhibitors was also prepared as a control group (see Fig. S1 in the supplemental material). The initial NaCl concentrations in the ganjang samples—measured using the Mohr method—were approximately 14.3% ± 0.4% (wt/vol), and these remained fairly constant during the entire fermentation period. The initial pH of all ganjang samples was ∼6.6. Interestingly, the pH profiles of the ganjang samples were distinctly different while the meju bricks were present in the ganjang solution (decreasing to pH 4.4) and after the meju bricks were removed from the ganjang solution (increasing to pH 7.8) (Fig. 1a). These results suggest that the metabolic features or activities might be quite different between these states. However, the pH values of the ganjang samples treated with microbial inhibitors slowly decreased while the meju bricks were present in the ganjang solution, and these values were relatively constant after the meju bricks were removed; this phenomenon might be attributed the release of organic acids from the meju bricks.

10.1128/mSystems.00441-21.1 FIG S1 The schematic outline for the preparation, sampling, and analyses of ganjang samples carried out in this study. The methods or programs used for the analyses were indicated beside the arrows. Download FIG S1, DOCX file, 0.1 MB. Copyright © 2021 Chun et al. 2021 Chun et al. https://creativecommons.org/licenses/by/4.0/ This content is distributed under the terms of the Creative Commons Attribution 4.0 International license.

Quantitative real-time PCR (qPCR) revealed that bacterial and fungal abundances increased from the initial values of approximately 1.0 × 10^6^ and 1.0 × 10^4^ copies/ml (day 0) to approximately 4.8 × 10^7^ and 4.6 × 10^5^ copies/ml (60 days), respectively, while the meju bricks were present in the ganjang solution; however, these values rapidly decreased after the meju bricks were removed from the ganjang solution (90 days) (Fig. 1b). Generally, the numbers of bacterial 16S rRNA gene copies were approximately 10^2^ times higher than those of fungal internal transcribed spacer (ITS) gene copies in the ganjang samples. The bacterial and fungal abundances of the ganjang solution treated with microbial inhibitors steadily and slowly decreased during the entire fermentation period. In addition, when the ganjang samples treated with microbial inhibitors were spread on agar medium, no bacterial or fungal colony growth was observed (data not shown). These results suggest that microbial inhibitors successfully inhibited microbial growth in the ganjang.

**FIG 2:**
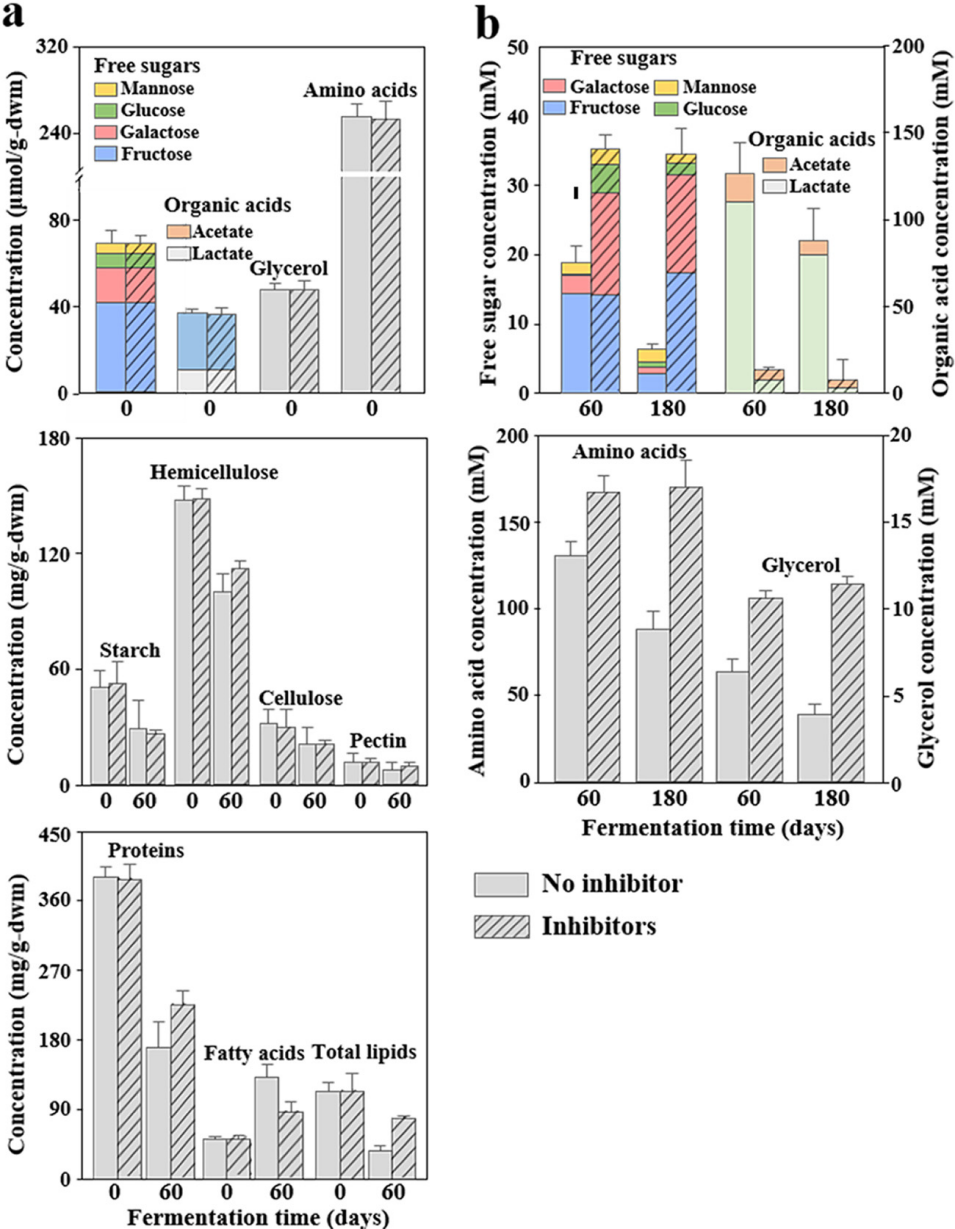
Major compounds present in the meju bricks (a) and ganjang solution (b) with and without microbial inhibitors. The compound content in the meju bricks was measured before adding the meju bricks into the brine solution (0 day) and after removing the meju bricks from the ganjang solution with and without microbial inhibitors (60 days). The error bars indicate the standard errors, measured in triplicate. g-dwm, g (dry weight) meju.

Compound analysis of the meju bricks revealed that considerable amounts of free sugars, organic acids, glycerol, and amino acids—which might be produced during a precedent fermentation of meju bricks—were already present in the meju bricks (Fig. 2a). Fructose was the most abundant free sugar, and galactose, glucose, and mannose were identified as the other major free sugars. Acetate and lactate were detected as major organic acids in the meju bricks, with acetate being more abundant. The content of macromolecules, including polysaccharides, proteins, and total lipids in the meju bricks, decreased while the meju bricks were present in the ganjang solution. However, the macromolecule content in the ganjang samples at 60 days was very low (data not shown), suggesting that the decrease in the meju macromolecule content through release into the ganjang solution might be negligible. The decrease in starch, cellulose, and pectin in the meju bricks treated with microbial inhibitors was relatively similar to that of the meju bricks without microbial inhibitors while the meju bricks were present in the ganjang solution. However, the decrease in hemicellulose, proteins, and total lipids (the increase of fatty acids) in the meju bricks without microbial inhibitors was significantly greater than that in the meju bricks treated with microbial inhibitors, suggesting that microbes also contribute to their degradation.

The concentrations of free sugars, amino acids, and glycerol in the ganjang solutions increased rapidly during the initial 60 days, and their increases in the ganjang without microbial inhibitors were significantly less than those in the ganjang treated with microbial inhibitors (Fig. 2b). After the meju bricks were removed from the ganjang solution (60 days), the concentrations of these compounds significantly decreased in the ganjang solution without microbial inhibitors, whereas the levels were almost constant in the ganjang solution treated with microbial inhibitors. These results suggest that considerable amounts of free sugars, amino acids, and glycerol may be consumed by microbial metabolism during ganjang fermentation. Conversely, the concentrations of organic acids at day 60 were much higher in ganjang samples without microbial inhibitors than those in ganjang samples with microbial inhibitors, suggesting that organic acids might be produced through microbial fermentation. In addition, the concentrations of organic acids also decreased significantly, although organic acids can be produced from free sugars, after the meju bricks were removed from the ganjang solution, which suggests that the consumption of organic acids by microbial metabolism was actively occurring during ganjang fermentation. However, lactate was the major organic acid component in the ganjang solution, whereas acetate was the major organic acid component in the meju bricks, suggesting that lactate fermentation is the major fermentation process during ganjang fermentation, whereas acetate fermentation is the major fermentation process during meju fermentation.

### Bacterial and fungal communities during ganjang fermentation.

The bacterial and fungal communities of ganjang samples, raw materials (meju bricks and solar salts), and 60-day meju bricks were investigated through an amplicon-based analysis. In total, 34,513 and 69,746 high-quality bacterial 16S rRNA and fungal ITS gene sequencing reads were obtained, and all samples reached almost complete coverage (∼100% Good’s coverage; see Fig. S2 in the supplemental material). Diversity analyses indicated that bacterial and fungal diversities generally increased as the fermentation progressed (see Table S1 in the supplemental material).

10.1128/mSystems.00441-21.2 FIG S2 Estimated Good’s coverages of bacterial 16S rRNA gene, fungal ITS2 gene, metagenome, and metatranscriptome sequencing reads used for the bioinformatic analyses in this study. Download FIG S2, DOCX file, 0.04 MB. Copyright © 2021 Chun et al. 2021 Chun et al. https://creativecommons.org/licenses/by/4.0/ This content is distributed under the terms of the Creative Commons Attribution 4.0 International license.

10.1128/mSystems.00441-21.6 TABLE S1 Summary of the bacterial and fungal sequencing data of the ganjang samples during fermentation and their statistical diversity indices. M and S represent meju bricks and solar salts used for the ganjang preparation. M_60_ represents sequencing data of meju bricks removed from the ganjang solution after 60 days. Download Table S1, DOCX file, 0.02 MB. Copyright © 2021 Chun et al. 2021 Chun et al. https://creativecommons.org/licenses/by/4.0/ This content is distributed under the terms of the Creative Commons Attribution 4.0 International license.

**FIG 3:**
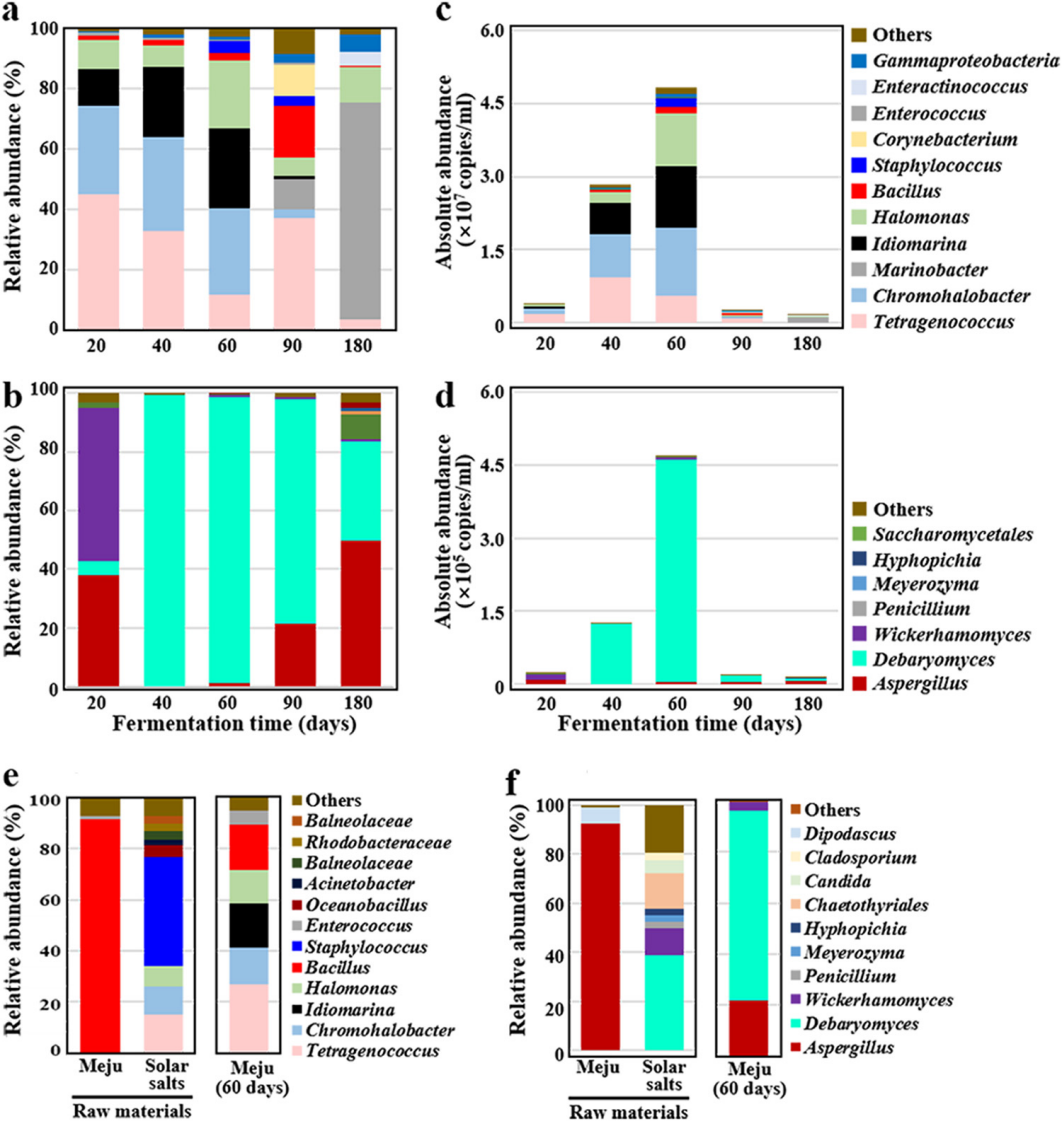
Relative abundances of bacteria (a) and fungi (b), based on 16S rRNA and ITS gene sequences, respectively, in the ganjang during fermentation. Absolute abundances of bacteria (c) and fungi (d) were estimated by multiplying the relative bacterial and fungal abundances by the mean values of the 16S rRNA and ITS gene copies, respectively, shown in Fig. S1B. Bacterial (e) and fungal (f) communities in the raw materials (meju bricks and solar salts) used for the ganjang preparation and those in the meju bricks removed from the ganjang solution after 60 days were also analyzed. “Others” comprises bacterial or fungal genera with <3.0% prevalence in all samples.

Bacterial community analysis revealed that Tetragenococcus, Chromohalobacter, Idiomarina, and Halomonas were the major genera present in the ganjang samples while the meju bricks were present in the ganjang solution (Fig. 3a). After the meju bricks were removed from the ganjang solution, *Tetragenococcus* and *Halomonas* were still abundant, but the relative abundances of *Chromohalobacter* and *Idiomarina* were found to be very low, and instead, *Marinobacter* and *Corynebacterium* were found to be abundant. Bacillus, which has previously been shown to be a major bacterial group present during meju fermentation (20), was identified over the entire fermentation time, but its relative abundance was generally low. Fungal community analysis revealed that Debaryomyces was the most abundant group during the entire fermentation period, especially after 40 days (Fig. 3b). Aspergillus, which has been shown to be a major fungal group present during meju fermentation (20), was also identified as a major fungus during the early fermentation period (20 days), but its relative abundance was very low at 40 and 60 days. However, Aspergillus became dominant again after the meju bricks were removed from the ganjang solution. *Wickerhamomyces* was also identified at a high relative abundance during the early fermentation period (20 days); however, after this period, its relative abundance was very low.

Microbial community analysis of raw materials (meju bricks and solar salts) used for ganjang fermentation showed that *Bacillus* and Aspergillus were predominant in meju bricks, whereas halotolerant or halophilic microbes such as *Tetragenococcus*, *Idiomarina*, *Chromohalobacter*, *Halomonas*, Staphylococcus, *Debaryomyces*, and *Wickerhamomyces* that were identified as being abundant in the ganjang samples were the dominant bacteria in solar salts (Fig. 3e and f). Principal-component analysis (PCA) based on the bacterial and fungal communities also revealed that solar salts were more closely grouped than meju bricks with ganjang samples (see Fig. S3 in the supplemental material). These results suggest that solar salts are the major source of microbes playing important roles during ganjang fermentation, as previously reported (21). The bacterial and fungal communities in the meju bricks after 60 days of fermentation were shown to be relatively similar to those in the ganjang samples, despite the fact that *Bacillus* and Aspergillus—probably derived from meju bricks of raw materials—were still abundant (Fig. 3e and f). The PCA results also indicated that the microbial communities of the 60-day meju bricks were similar to those of the ganjang samples (Fig. S3).

10.1128/mSystems.00441-21.3 FIG S3 Principal component analysis of bacterial (a) and fungal (b) communities of ganjang samples, raw materials (M, meju bricks; S, solar salts), and 60-day meju bricks (M_60_). Numbers beside the closed circles indicate the fermentation time (days) and M_60_ represents microbial communities in meju bricks removed from ganjang solution after 60 days. Download FIG S3, DOCX file, 0.04 MB. Copyright © 2021 Chun et al. 2021 Chun et al. https://creativecommons.org/licenses/by/4.0/ This content is distributed under the terms of the Creative Commons Attribution 4.0 International license.

The absolute abundances of each microbial taxon were estimated by multiplying the relative abundances of the bacterial and fungal genera by their numbers of 16S rRNA and ITS gene copies (Fig. 1b), respectively. The absolute abundance analysis revealed that *Tetragenococcus*, *Chromohalobacter*, *Idiomarina*, and *Halomonas* were highly abundant (Fig. 3c), suggesting that they may be in large part responsible for ganjang fermentation. However, the abundances of *Marinobacter*, *Bacillus*, and *Corynebacterium*, which showed high relative abundances after the meju bricks were removed from the ganjang solution, were low, suggesting that these bacteria may be less responsible for ganjang fermentation. *Debaryomyces* was identified as the most highly abundant fungal group at 40 and 60 days, whereas Aspergillus and *Wickerhamomyces*, which showed high relative abundances during the early or late fermentation periods, showed very low abundances over the entire ganjang fermentation period (Fig. 3d). These results suggest that the relative abundance of microbes does not represent the extent of contribution to overall ganjang fermentation because even though relative microbial abundances are high during ganjang fermentation, their absolute abundances can be very low.

In total, 19.2 Gb of metagenomic reads were obtained from five ganjang sampling times with an average Good’s coverage of 95.2% (Fig. S2), and metagenomic assemblies were performed for each sample’s data and all combined data, generating 15 dereplicated metagenome-assembled genomes (MAGs) (see Table S2 in the supplemental material). No MAG corresponding to Aspergillus was generated, probably due to the low levels of Aspergillus sequences in the metagenomic reads. Among the 15 MAGs, 10 were of high quality, with ≥90% completeness and a ≤5% contamination rate; the remaining 5 MAGs were of poor quality. Therefore, the isolation of bacterial and fungal strains corresponding to low-quality MAGs from the ganjang samples was attempted. Bacterial and fungal strains corresponding to all MAGs, except for *Corynebacterium*, Alteromonadaceae, Idiomarinaceae, and Micrococcaceae, were successfully isolated, and an Aspergillus strain was also isolated. The genomes of four bacterial strains (two *Tetragenococcus*, one *Chromohalobacter*, and one *Marinobacter*) and three fungal strains (one each of *Debaryomyces*, *Wickerhamomyces*, and Aspergillus) were sequenced and used for further studies, together with 10 high-quality MAGs (Table 1). The isolate genomes and MAGs were representative of the genomes of the major genera identified from the amplicon-based community analysis.

**TABLE 1:** General information on the 17 microbial genomes used for the metagenomics and metatranscriptomic analyses and pathway reconstructions of the ganjang microbiota

Genome name	Source^*a*^	Genome size (Mb)	G+C content (%)	GenBank accession no.	No. of 16S rRNA or ITS genes (related genome GenBank accession no.)^*b*^
Bacillus sp. KG1	M	3.1	46.7	JABXGZ000000000	10 (CP011939)
*Bacillus* sp. KG2	M	3.1	37.6	JABXHA000000000	8 (CP023704)
*Bacillus* sp. KG3	M	4.5	45.5	JABXHB000000000	9 (CP021920)
Corynebacterium sp. KG4	M	3.0	68.9	JABXHC000000000	5 (CP009251)
Staphylococcus sp. KG5	M	3.1	32.9	JABXHD000000000	6 (CP017466)
Halomonas sp. KG6	M	3.0	64.7	JABXHE000000000	4 (CP019326)
Virgibacillus sp. KG7	M	3.6	36.5	JABXHF000000000	8 (CP017962)
Alteromonadaceae sp. KG8	M	4.9	54.5	JABXHG000000000	3 (CP011929)
Idiomarinaceae sp. KG9	M	4.2	46.5	JABXHH000000000	4 (CP027188)
Micrococcaceae sp. KG10	M	3.1	56.5	JABXHI000000000	6 (NC_018531)
Tetragenococcus sp. KG11	I	2.4	36.0	JABEVO000000000	5 (CP012047)
*Tetragenococcus* sp. KG12	I	2.5	35.7	JABTEK000000000	5 (CP012047)
Chromohalobacter sp. KG13	I	3.5	61.0	JABEVP000000000	5 (NC_007963)
Marinobacter sp. KG14	I	3.5	55.0	JABEVQ000000000	3 (NC_017067)
Aspergillus sp. KG15	I	38.0	47.8	JABULI000000000	3 (NC_036441)
Wickerhamomyces sp. KG16	1	23.9	34.5	JAHTLX000000000, JAHTLY000000000	1
Debaryomyces sp. C11	1	24.5	36.4	JADOBC000000000, JADOBD000000000	5

a M, metagenome-assembled genome; I, isolate-derived genome.

b Where the exact numbers of the 16S rRNA and ITS genes could not be obtained from the genomes, they were estimated from the most closely related complete genome sequences available in GenBank (shown in parenthesis).

10.1128/mSystems.00441-21.7 TABLE S2 General information on the metagenome-assembled genomes (MAGs) obtained through a binning process of the metagenome sequences derived from the ganjang samples. Download Table S2, DOCX file, 0.02 MB. Copyright © 2021 Chun et al. 2021 Chun et al. https://creativecommons.org/licenses/by/4.0/ This content is distributed under the terms of the Creative Commons Attribution 4.0 International license.

**FIG 4:**
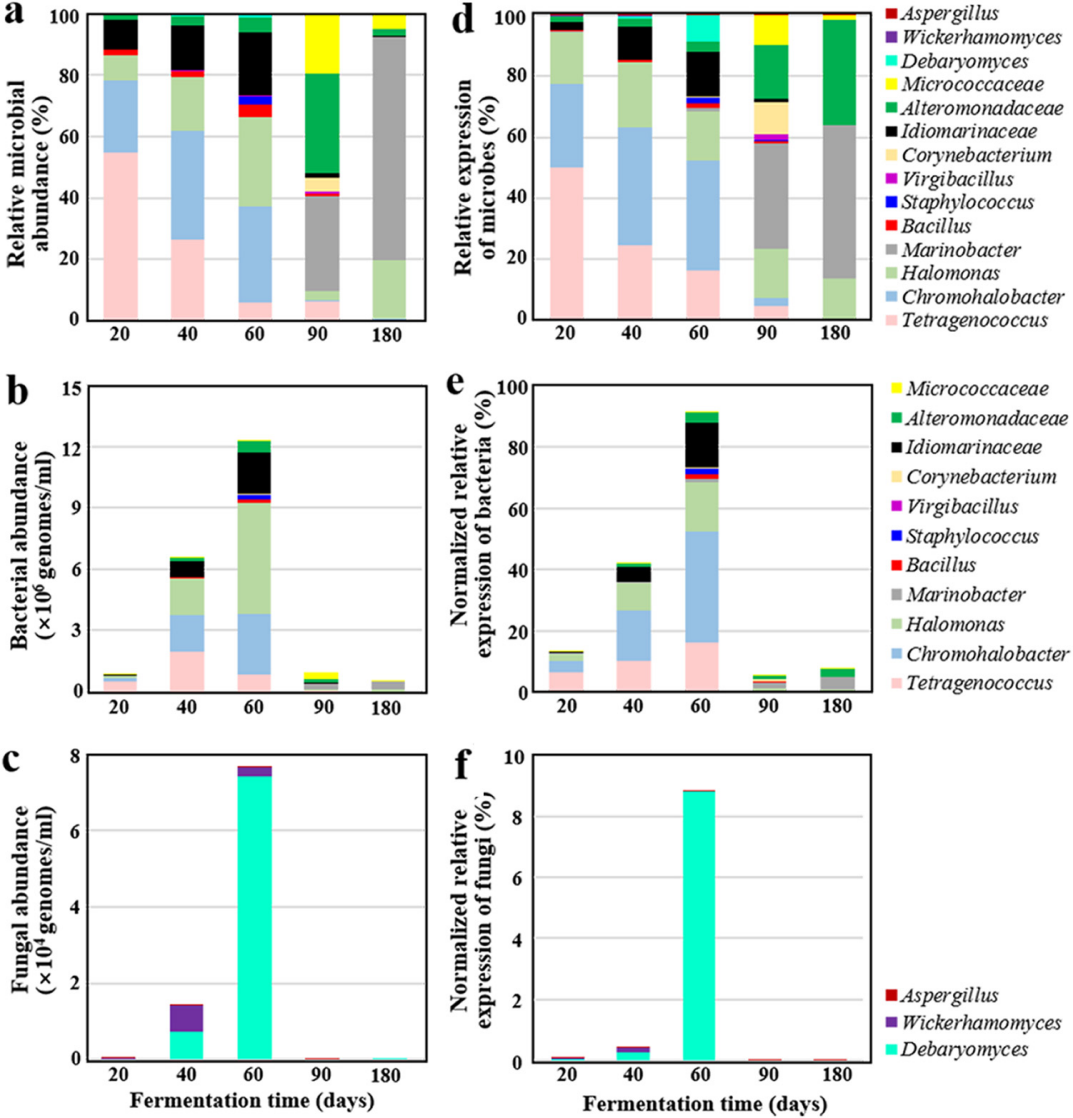
Metagenomic abundances (a, b, and c) and metatranscriptomic expressions (d, e, and f) of microbes in the ganjang solution during fermentation. The relative microbial abundances (a) and transcriptional expressions (d) were calculated based on the numbers of metagenomic sequencing and metatranscriptional mRNA reads mapped to the 17 representative genomes, respectively; the relative microbial abundances were normalized based on their genome sizes. Bacterial (b) and fungal (c) abundances were calculated by multiplying the relative microbial abundances shown in panel a by the mean values of their gene copies, shown in Fig. S1b in the supplemental material, and then dividing by the copy numbers of the 16S rRNA or ITS genes in each genome. The expressions of bacteria (e) and fungi (f) were normalized based on the sequenced read numbers of spike-in RNA in each sample and indicated in a relative manner based on the total expression of the bacteria and fungi at 60 days.

For the metagenome-based community analysis, approximately 3.6 to 7.5 million high-quality metagenomic reads for each sample were mapped onto the genomes of the 7 isolates and 10 MAGs, corresponding to 91.5 to 99.3% reads (see Table S3 in the supplemental material), suggesting that the 17 genomes were representative of the microbiota present during ganjang fermentation. Read numbers mapped onto each genome were normalized based on their genome sizes and indicated in a relative manner (Fig. 4). The mapped profiles were generally similar to the community profiles obtained by the amplicon-based analysis (Fig. 4b and c). Fungal abundances (primarily represented by *Debaryomyces* and *Wickerhamomyces*) were also less than 1% of bacterial abundances; this was similar to the results of the amplicon-based analysis. However, the abundance of *Wickerhamomyces* was much higher than that in the amplicon-based analysis (Fig. 4c), suggesting that *Wickerhamomyces* may play an important role during ganjang fermentation. The abundance of Aspergillus, which was relatively high during the early and late fermentation periods in the amplicon-based analysis, was very low during the entire ganjang fermentation period, suggesting that Aspergillus is not a key microbe during ganjang fermentation.

10.1128/mSystems.00441-21.8 TABLE S3 Summary of the metagenomic sequencing data of the ganjang samples. Download Table S3, DOCX file, 0.02 MB. Copyright © 2021 Chun et al. 2021 Chun et al. https://creativecommons.org/licenses/by/4.0/ This content is distributed under the terms of the Creative Commons Attribution 4.0 International license.

### Metatranscriptomic analysis of ganjang fermentation.

Approximately 33.4 to 71.3 million high-quality mRNA sequencing reads for each sample were obtained with an average Good’s coverage of 98.4% (Fig. S2) and mapped onto the isolate genomes and MAGs. Then, 78.4 to 88.6% of reads were mapped onto the genomes (see Table S4 in the supplemental material). The relative expression of each genus was generally similar to the corresponding relative abundance obtained by the metagenome-based community analysis, but the relative expression of some taxa was somewhat different from the corresponding relative abundance in the community analysis (Fig. 4d). *Tetragenococcus*, *Chromohalobacter*, *Halomonas*, *Idiomarinaceae*, *Marinobacter*, *Corynebacterium*, and *Alteromonadaceae*, which were identified as being abundant in the metagenome-based community analysis, also exhibited a generally high relative expression. However, the relative expression of some taxa, such as *Halomonas* (at 60 and 90 days), *Idiomarinaceae* (at 90 days), and *Micrococcaceae* and *Alteromonadaceae* (at 90 and 180 days) was quite different from the corresponding relative abundance in the metagenome-based community analysis, suggesting that their abundances and activities during these periods might be different. The transcriptional levels of these yeasts amounted to more than 8% of the total expression data, although their relative abundances were less than 1% in the community analyses, suggesting that fungal genes may be highly expressed compared to bacterial genes (22). The normalized transcriptional expression based on the spike-in RNA sequencing in each sample showed that the transcriptional levels in the ganjang microbiome were very high while the meju bricks were present in the ganjang solution but became very low after the meju bricks were removed (Fig. 4e and f), similar to the microbial abundance levels (Fig. 3c and d). The metatranscriptomic analysis showed that *Debaryomyces* was identified as a yeast with predominantly transcriptional expression (Fig. 4f), suggesting that *Debaryomyces* may be yeast playing important roles during ganjang fermentation. However, the transcriptional level of *Wickerhamomyces* was very low with respect to its abundance in the metagenome-based community analysis (Fig. 4c and f), suggesting that its metabolic activity might be low during ganjang fermentation. The transcripts of Aspergillus were also rarely identified over the entire fermentation period, suggesting that Aspergillus may not play a very important role during ganjang fermentation, although it was abundantly identified in the fungal community analysis (Fig. 3b).

10.1128/mSystems.00441-21.9 TABLE S4 Summary of the metatranscriptomic sequencing data of the ganjang samples. Download Table S4, DOCX file, 0.02 MB. Copyright © 2021 Chun et al. 2021 Chun et al. https://creativecommons.org/licenses/by/4.0/ This content is distributed under the terms of the Creative Commons Attribution 4.0 International license.

The metabolic features of the ganjang microbiome during fermentation were investigated through the functional Kyoto Encyclopedia of Genes and Genomes (KEGG) classification of the mRNA reads mapped onto the isolate genomes and MAGs (see Table S5 in the supplemental material). Genes related to carbohydrate and energy metabolism at the secondary level and glycolysis/gluconeogenesis; fructose and mannose metabolism; galactose metabolism; pentose phosphate pathway; and pyruvate metabolism at the tertiary level were highly upregulated during the early fermentation periods, and their transcriptional expression rapidly decreased as the fermentation progressed (see Fig. S4 in the supplemental material). The transcriptional profile of the phosphotransferase system associated with carbohydrate transports showed a similar pattern. However, the transcriptional expression of genes associated with other types of metabolism, such as lipid and amino acid metabolism, was found to be relatively constant over the entire fermentation period. The transcriptional expression of genes associated with cell growth generally decreased as the fermentation progressed. These results suggest that carbohydrate metabolism involving free sugars, such as fructose, glucose, galactose, and mannose, was the major metabolic pathways during ganjang fermentation, with the activities decreasing as free sugar concentrations decreased. The transcription of genes related to signal transduction, cell motility, bacterial secretion system, and two-component systems increased at day 180, which might be associated with the increase in environmental stresses such as nutrient depletion at that time point (23).

10.1128/mSystems.00441-21.4 FIG S4 Transcriptional expression of representative KEGG functional categories at the secondary (a) and tertiary (b) levels during ganjang fermentation. The RPKM (read numbers per kilobase of each coding sequence, per million mapped reads) values on the Y axes were calculated as the sums of the RPKM values of all ganjang microbes belonging to the same KEGG functional categories. Download FIG S4, DOCX file, 0.1 MB. Copyright © 2021 Chun et al. 2021 Chun et al. https://creativecommons.org/licenses/by/4.0/ This content is distributed under the terms of the Creative Commons Attribution 4.0 International license.

10.1128/mSystems.00441-21.10 TABLE S5 Information of genes in the 17 genomes of ganjang major microbiota and their transcriptional expressions during ganjang fermentation. Download Table S5, XLSX file, 5 MB. Copyright © 2021 Chun et al. 2021 Chun et al. https://creativecommons.org/licenses/by/4.0/ This content is distributed under the terms of the Creative Commons Attribution 4.0 International license.

The metabolic features of the ganjang microbiome were also investigated further by mapping the normalized bacterial and fungal mRNA transcripts onto the KEGG pathways of the 17 representative genomes (see Fig. S5 in the supplemental material). The overall transcription of genes associated with metabolic pathways increased as the fermentation progressed while the meju bricks were present in the ganjang solution, but this quickly decreased after the meju bricks were removed; these results were similar those obtained for microbial abundance and transcriptions (Fig. 3 and 4). In particular, while the meju bricks were present in the ganjang solution, genes related to the bacterial pathways associated with carbohydrate, amino acid, lipid, and nucleotide metabolisms and the fungal pathways associated with carbohydrate and amino acid metabolisms were highly expressed, and the transcriptional expression of genes related to carbohydrate metabolism greatly decreased after the meju bricks were removed from the ganjang solution.

10.1128/mSystems.00441-21.5 FIG S5 Transcriptional expression of the metabolic pathways of bacteria (red) and fungi (blue) at 20 (a), 40 (b), 60 (c), 90 (d) and 180 (e) days of ganjang fermentation. The KEGG metabolic pathways were generated using the iPath v3 module based on the KEGG Orthology numbers of the metabolic genes identified from the 17 microbial genomes listed in Table 1, and their transcriptional levels are quantitatively depicted using different line thickness and color brightness, based on the normalized RPKM (read numbers per kilobase of each coding sequence, per million mapped reads) values derived from the sequenced read numbers of spike-in RNA in each sample. Download FIG S5, DOCX file, 1.1 MB. Copyright © 2021 Chun et al. 2021 Chun et al. https://creativecommons.org/licenses/by/4.0/ This content is distributed under the terms of the Creative Commons Attribution 4.0 International license.

### Metatranscriptomic analyses of metabolic pathways during ganjang fermentation.

**FIG 5:**
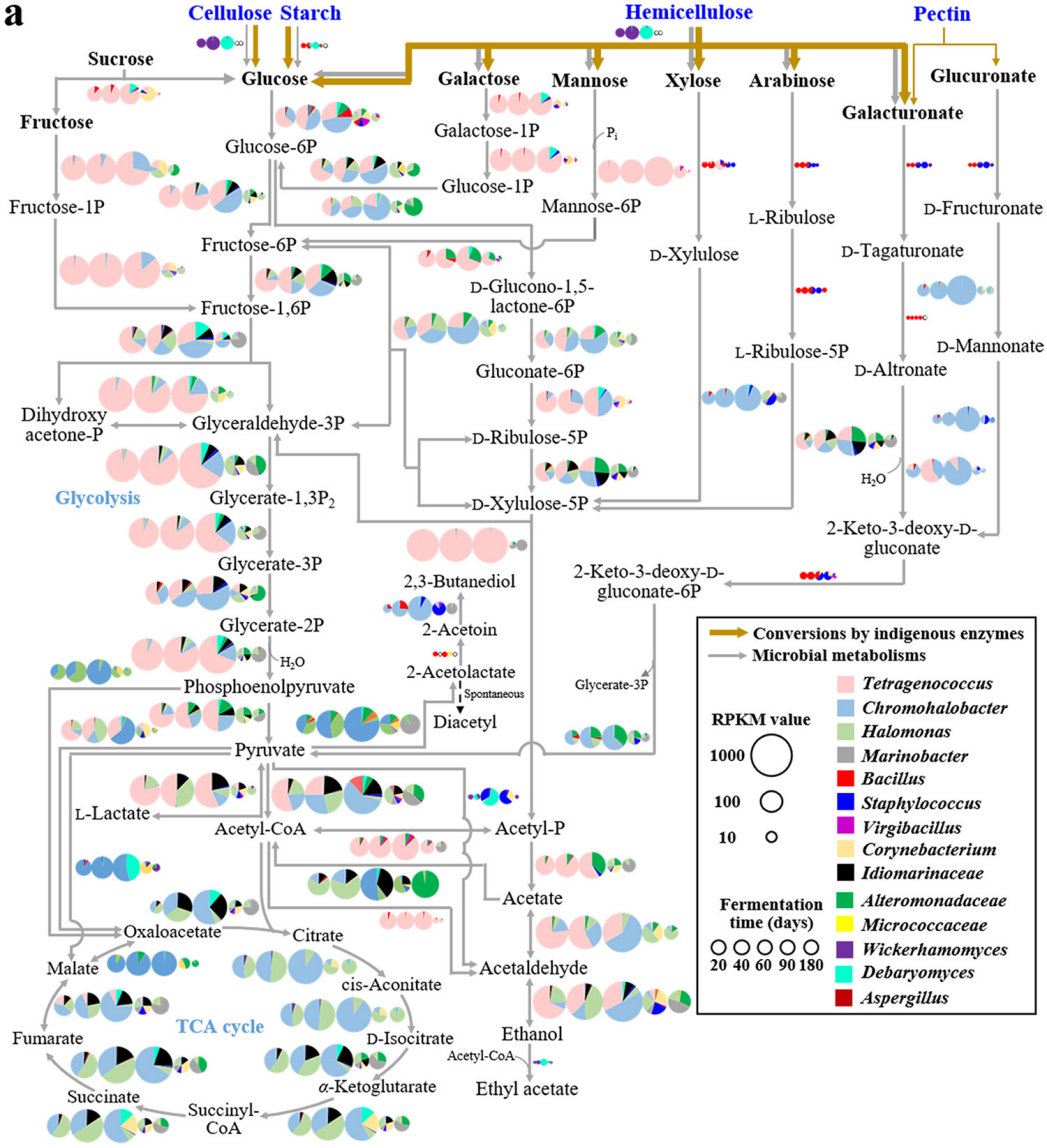

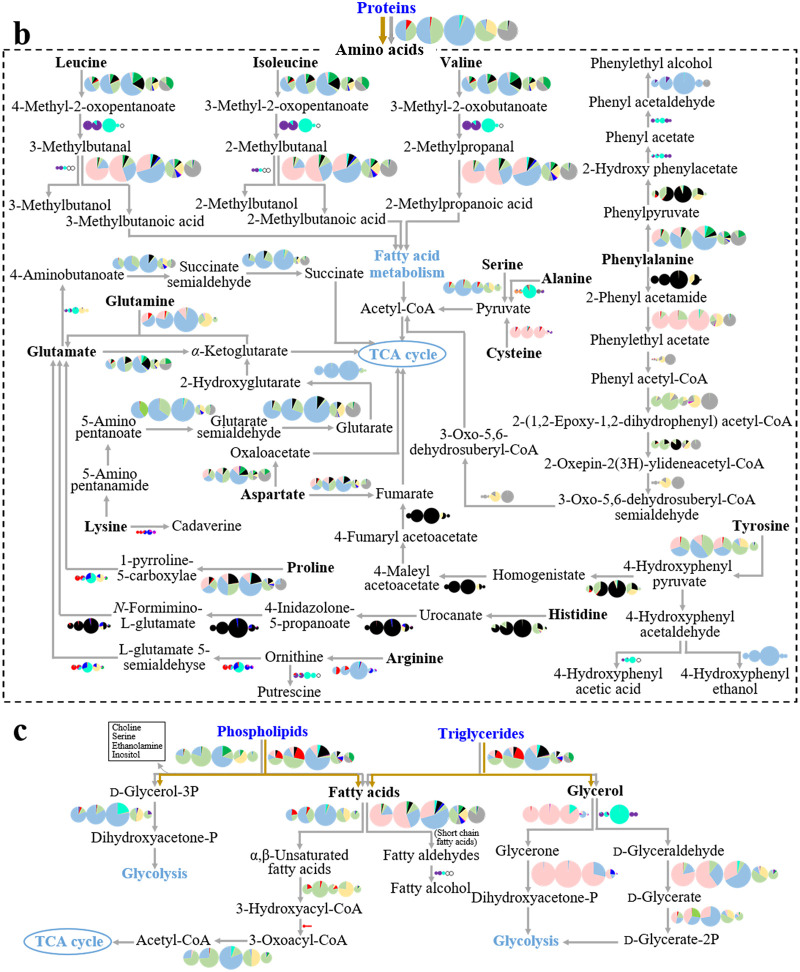
Proposed metabolic pathways of the ganjang microbiome for carbohydrates (a), amino acids (b), and lipids (c) and their transcriptional expression during fermentation (20, 40, 60, 90, and 180 days). The degradation of polysaccharides (a), proteins (b), and lipids (c) by indigenous enzymes is arbitrarily depicted using lines of different thicknesses based on their contributions, shown in Fig. 1. Transcriptional levels of the ganjang microbiota are visualized using pie charts based on the normalized read numbers per kilobase of each coding sequence per million mapped reads (RPKM) values of the functional genes in each ganjang microbe. The detailed names and expressional levels of the metabolic genes are described in Table S1 in the supplemental material.

For a more scrutinized analysis of the metabolic features of the ganjang microbiome during fermentation, the carbohydrate, protein, and lipid metabolic pathways for the 17 representative genomes were reconstructed, and the expression of metabolic genes was transcriptionally analyzed (Fig. 5). Transcriptional analysis revealed that the degradation of carbohydrate polymers, including cellulose, starch, and hemicellulose, in the meju bricks, might be attributed primarily to the yeasts *Wickerhamomyces* (20 and 40 days) and *Debaryomyces* (60 days), while the meju bricks were present in the ganjang solution (Fig. 5a). *Bacillus* also might contribute to starch degradation during the early fermentation period. Metabolic pathway and transcriptional analysis for carbohydrates revealed that major free sugars, including fructose, glucose, galactose, and mannose, (in particular, fructose, galactose, and mannose), might have been metabolized in large part by *Tetragenococcus* while the meju bricks were present in the ganjang solution. The analysis showed that *Chromohalobacter*, *Halomonas*, *Marinobacter*, *Idiomarinaceae*, and Alteromonadaceae species might be also responsible for the metabolism of the major free sugars, especially after the meju bricks were removed from the ganjang solution. Minor carbohydrates, such as xylose, arabinose, galacturonate, and glucuronate might be metabolized in large part by *Chromohalobacter*, but they are likely first converted by *Bacillus* and Staphylococcus into intermediates metabolizable by *Chromohalobacter*.

The metabolic pathway and transcriptional analyses also revealed that *Tetragenococcus* harbored the complete glycolysis pathway, the 6-phosphogluconate/phosphoketolase pathway, and pyruvate pathways producing lactate, acetate, and ethanol (24). These pathways were highly expressed while the meju bricks were present in the ganjang solution (Fig. 5a). *Tetragenococcus* did not harbor a complete tricarboxylic acid (TCA) cycle, and the phosphoketolase (xylulose-5P + P*i* ↔ glyceraldehyde-3P + acetyl-*P* + H_2_O) gene, a specific gene for heterolactic fermentation, was predominantly expressed. These results suggested that in *Tetragenococcus*, a facultative heterolactic fermentation pathway may be involved in the production of lactate, acetate, and ethanol from the major free sugars. *Chromohalobacter*, *Halomonas*, *Marinobacter*, *Idiomarinaceae*, and *Alteromonadaceae* species also harbored metabolic pathways associated with lactate, acetate, and ethanol, and expressed metabolic genes at high levels. However, these bacteria showed high transcriptional expression of genes involved in the glycolysis pathway, the pentose phosphate pathway, and the TCA cycle, suggesting that these bacteria perform aerobic metabolism and not fermentation. NADH dehydrogenase, succinate dehydrogenase, cytochrome *bd* oxidase, and cytochrome *bo* oxidase genes associated with aerobic respiration were also highly expressed (Table S5). These results suggest that these bacteria might perform aerobic metabolism, consuming lactate, acetate, and ethanol produced by *Tetragenococcus*, and not fermentation, which would have resulted in the production of lactate, acetate, and ethanol from free sugars. The metabolic pathway and transcriptional analyses also showed that diacetyl and acetoin, responsible for cheese flavors, might be produced mainly by bacteria, whereas ethyl acetate, a molecule responsible for floral scent, might be produced mainly by yeasts, namely *Wickerhamomyces* and *Debaryomyces*.

Transcriptional analysis of the genes coding for proteinases revealed that proteins might be degraded to amino acids mainly by *Chromohalobacter* and *Halomonas* during ganjang fermentation (Fig. 5b). Transcriptional analyses of genes associated with protein metabolism revealed that amino acids might be metabolized by diverse microorganisms, mainly bacteria, and the major metabolic microbes were different depending on the amino acids or the fermentation period. *Chromohalobacter*, *Halomonas*, *Idiomarinaceae*, and *Tetragenococcus* might be responsible in large part for amino acid metabolism while the meju bricks were present in the ganjang solution, whereas *Chromohalobacter*, *Marinobacter*, and *Corynebacterium* might be responsible in large part for amino acid metabolism after the meju bricks were removed. The analyses revealed that branched and aromatic amino acids, such as leucine, isoleucine, valine, and phenylalanine, might be first metabolized to their corresponding intermediates, mainly by bacteria, and then converted to ganjang-flavoring compounds, such as methylbutanal, methylbutanol, methylpropanol, phenyl acetate, and phenyl acetaldehyde (25) by yeasts (*Wickerhamomyces* and *Debaryomyces*), which suggests that yeasts may be responsible in large part for producing flavoring compounds during ganjang fermentation. The analyses also showed that the levels of flavoring compounds produced by yeasts might decrease through further metabolisms by bacteria such as *Chromohalobacter* and *Tetragenococcus*. However, phenylethyl alcohol and 4-hydroxyphenyl ethanol, other flavoring compounds of ganjang, might be produced through further metabolism of phenyl acetaldehyde and 4-hydroxyphenyl acetaldehyde by *Chromohalobacter*. In traditionally fermented soybean foods, biogenic amines (BAs) are frequently produced through the decarboxylation of certain amino acids (9, 26). Transcriptional analyses showed that cadaverine and putrescine might be produced through the decarboxylation of lysine and ornithine by *Bacillus*, Staphylococcus, or Debaryomyces during ganjang fermentation. Additionally, transcriptional analyses of genes related to metabolic pathways revealed that amino acids might be completely metabolized through the TCA cycle, causing the decrease in amino acid levels during ganjang fermentation.

Transcriptional analysis of genes encoding lipases (or esterases) showed that lipids (triglycerides and phospholipids) might be hydrolyzed to glycerol, fatty acids, and glycerol-3P by various bacteria, such as *Chromohalobacter*, *Halomonas*, and *Bacillus* (Fig. 5c). Transcriptional analyses of genes related to lipid metabolic pathways revealed that glycerol might be metabolized mainly by *Tetragenococcus*, whereas fatty acids and glycerol-3P might be metabolized mainly by *Chromohalobacter* and *Halomonas*. These results suggest that glycerol might be used primarily as a substrate for lactate fermentation, whereas fatty acids and glycerol-3P might be completely metabolized primarily through aerobic respiration. The analyses also showed that short-chain fatty acids could be converted into fatty alcohols, the major flavoring compounds of ganjang, from fatty aldehydes produced by bacteria—such as *Tetragenococcus* and *Chromohalobacter*— and yeasts while the meju bricks were present in the ganjang solution.

## DISCUSSION

In Korea, ganjang fermentation is carried out by soaking meju bricks (fermented soybeans) in a high concentration of solar salt solution without sterilization (1). Meju bricks are removed from the ganjang solution after approximately 2 months, and the remaining ganjang solution is additionally fermented. During ganjang fermentation, removing the meju bricks from the ganjang solution is an important step that may greatly influence the metabolic features of ganjang microbiota because meju bricks are probably the sole nutrient source for ganjang microbiota. The pH profiles and microbial abundances (qPCR, metagenome, and metatranscriptome) indirectly representing the metabolic features and activities of ganjang microbiota in the ganjang solution were quite distinct before and after meju brick removal (Fig. 1 to 4). Figures 3 and 4 clearly suggest that microbial metabolism primarily occurs while meju bricks are present in the ganjang solution and that it significantly decreases after the removal of meju bricks.

It has been generally suggested that ganjang fermentation is processed through ganjang microbiota (1, 2, 6, 27). Our analysis also showed that *Tetragenococcus*-mediated heterotactic acid fermentation using free sugars and glycerol was the major fermentation process during ganjang fermentation (Fig. 2b and 5a). However, our metagenomic and metatranscriptomic analyses showed that aerobic or facultative aerobic bacteria such as *Chromohalobacter*, *Halomonas*, and *Marinobacter* were also abundant, and their metabolic activities were high during fermentation (Fig. 3 to 5), suggesting that aerobic metabolism may also be an important metabolic process influencing the taste and quality of ganjang. In particular, aerobic metabolism consuming organic compounds, including organic acids and amino acids, was more prevalent than fermentation, which results in the production of organic acids (Fig. 5), resulting in a decrease in the organic compound contents and an increase in the pH of the ganjang solution after the removal of meju bricks (Fig. 1a and 2b). The oxygen necessary for these aerobic metabolisms may be obtained from the surface of the ganjang solution. In addition, the decrease after 60 days in the content of polymeric compounds, such as cellulose, starch, proteins, and lipids, was not significantly different in the meju bricks regardless of microbial inhibition (Fig. 2), suggesting that in meju bricks, the polymeric compounds may be primarily degraded by indigenous enzymes, rather than by ganjang microbes. Nevertheless, metatranscriptomic analyses showed that halophilic yeasts such as *Debaryomyces* and *Wickerhamomyces* and halophilic bacteria such as *Chromohalobacter* and *Halomonas* might play important roles in the degradation of starch, cellulose, and hemicellulose (yeasts) and proteins and lipids (bacteria) (Fig. 5), suggesting that ganjang microbiota may also contribute to the degradation of polymeric compounds. The analysis of organic compounds showed that significant amounts of fatty acids, glycerol, organic acids, free sugars, and amino acids—which may be produced during meju fermentation, not ganjang fermentation—were already present in the meju bricks (Fig. 2), and they might be released into ganjang solution while meju bricks are present in the ganjang solution, suggesting that the fermentation quality of the meju bricks may also influence ganjang fermentation.

Previous studies have shown that *Bacillus* and Aspergillus, as well as halophilic microbes such as Staphylococcus, *Tetragenococcus*, *Chromohalobacter*, *Halomonas*, *Debaryomyces*, and *Wickerhamomyces* are abundantly present during ganjang fermentation, and it has been suggested that *Bacillus* and Aspergillus may be responsible in large part for ganjang fermentation (21, 28). Our relative community analysis showed that Aspergillus is the most abundant fungus during the early and late fermentation periods (Fig. 3b). However, our quantitative community, metagenome, and metatranscriptome analyses showed that *Bacillus* and Aspergillus were minor and metabolically inactive, and instead, halophilic or halotolerant microbes such as *Tetragenococcus*, *Chromohalobacter*, *Halomonas*, *Marinobacter*, *Debaryomyces*, and *Wickerhamomyces* were abundant and metabolically active during ganjang fermentation (Fig. 3 to 5). These results suggest that the halophilic or halotolerant microbes derived from solar salts, and not *Bacillus* and Aspergillus derived from meju bricks (Fig. 3e and f), may be majorly responsible for ganjang fermentation. In particular—although they are present at much lower abundances—yeasts such as *Debaryomyces* and *Wickerhamomyces* may be responsible in large part for the production of biogenic amines and flavors, thereby possibly affecting the quality and taste of ganjang.

**FIG 6:**
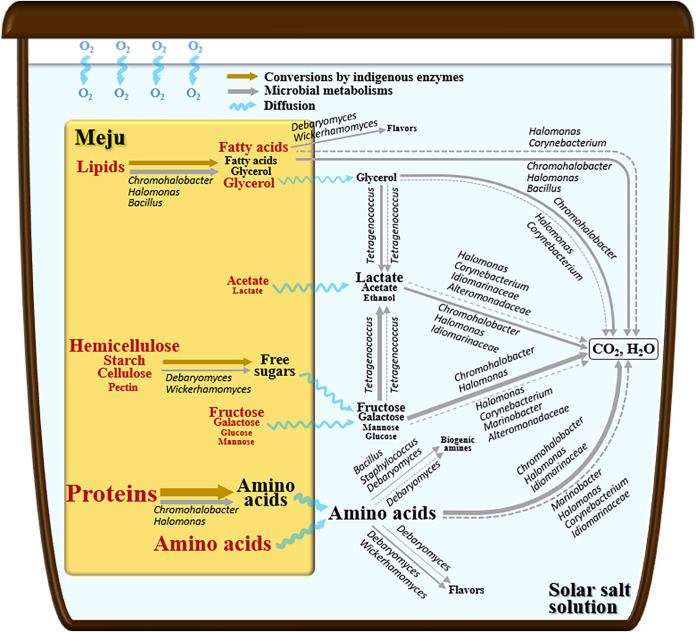
A schematic diagram of the metabolic networks of the ganjang microbiome during ganjang fermentation. Letter sizes indicate the arbitrary compound concentrations in the meju bricks and ganjang solution that were estimated from the metabolite data shown in Fig. 1. Pink and black letters represent compounds already present in meju bricks and metabolites produced during ganjang fermentation, respectively. Solid lines indicate metabolism occurring while meju bricks were present in the ganjang solution, whereas dotted lines indicate metabolism occurring after the meju bricks were removed from the ganjang solution, and their metabolic activities are displayed in a relative manner using different line thicknesses, based on the metabolism-associated transcriptomic expression shown in Fig. 5. The main ganjang microbes present in ganjang that are responsible for the metabolisms are indicated beside the lines.

Based on the combined results of major compound analysis, quantitative metagenome and metatranscriptome analyses, and metabolic pathway reconstruction and expressional analysis of major ganjang microbiota, we proposed a metabolic network of major ganjang microbiota during fermentation (Fig. 6). In this study, we showed that genome-centered metatranscriptomic analyses can provide a comprehensive and extensive understanding of the overall metabolic features of the ganjang microbiome during fermentation. This approach will also provide insights to attain a better understanding of various environmental microbial processes, including those involved in other food fermentation processes.

## MATERIALS AND METHODS

### Ganjang preparation and sampling.

Four batches of traditional Korean ganjang fermentation were prepared, as described previously, with some modifications (9). In brief, five meju bricks (approximately 5.5 kg) obtained from a manufacturer located in Haenam (Republic of Korea) were added to four Korean porcelain pots containing 20 liters of approximately 16% (wt/vol) solar salt (Shinan, Republic of Korea) solution. To serve as a negative control, sodium azide (0.5 g/liter) and fenhexamid (0.05 g/liter) were added to one of the four ganjang batches as inhibitors of bacterial and fungal growth, respectively. The ganjang batches were stored at room temperature (approximately 20 to 25°C), and their pH was periodically measured. After 60 days, the meju bricks were removed from the ganjang solution, and the remaining ganjang solution was then stored for 120 days. The liquid components (ganjang) were periodically sampled (0, 20, 40, 60, 90, and 180 days), and the solid components (meju) were sampled at 0 and 60 days. Ganjang and meju samples obtained from the three ganjang batches without inhibitors were pooled, and the meju samples were stored at −80°C. Ganjang samples were centrifuged for 5 min at 4°C (16,100 × *g*), and the pellets and supernatants were stored separately at −80°C. The schematic outline for the preparation, sampling, and analyses of ganjang fermentation is shown in Fig. S1 in the supplemental material.

### Chemical analyses and quantitative real-time PCR.

NaCl concentrations were measured according to the Mohr method (29). The cellulose, hemicellulose, starch, and pectin contents in the meju bricks were determined using Van Soest methods (30). The free sugars, amino acids, organic acids, and glycerol in the samples were analyzed using ^1^H nuclear magnetic resonance (NMR) spectroscopy, as described previously (9, 26). Total nitrogen compounds in the meju bricks were measured using the Kjeldahl method (31), and the protein content was calculated by subtracting the amino acid content from the total nitrogen compound content. Total lipids (triglycerides and phospholipids) and fatty acids were measured using Folch’s method (32). Bacterial and fungal abundances in the ganjang samples were estimated through qPCR using salmon sperm DNA (Sigma-Aldrich, USA) as the internal standard based on the copies of bacterial 16S rRNA and fungal internal transcribed spacer (ITS) genes, respectively, as described previously (33).

### Metagenomic DNA extraction and amplicon-based community analysis.

Genomic DNA was extracted from the ganjang samples, raw materials (meju bricks and solar salts), and meju bricks at 60 days using the FastDNA spin kit for soil (MP Biomedicals, USA), according to a previously described procedure (28). For amplicon-based community analysis, the V3-V4 region of bacterial 16S rRNA genes and the ITS2 region of fungal rRNA genes were PCR amplified and sequenced after pooling bacterial and fungal PCR products separately using a MiSeq paired-end platform (×300 bp; Illumina, USA) at Macrogen (Republic of Korea), as described previously (33). The Illumina MiSeq paired-end sequencing reads were processed to obtain high-quality bacterial and fungal sequencing reads for each sample and taxonomically classified, following the procedure described previously (33). Briefly, the sequencing reads were sorted into individual samples based on their barcode sequences. Adaptor sequences and low-quality reads were removed using Scythe and Sickle programs, respectively, and Good’s coverage was calculated using the Good’s coverage estimator available in Qiime 2. The bacterial and fungal sequencing reads were classified using Qiime 2, and PCA was performed based on their relative abundances using the “prcomp” function of R software (http://cran.r-project.org/). The absolute bacterial and fungal abundances in the ganjang during fermentation were estimated by multiplying the relative bacterial and fungal abundances by the numbers of bacterial and fungal gene copies obtained by qPCR.

### Metagenome sequencing and assembly.

Metagenomic DNA extracted from the ganjang samples was sequenced using an Illumina MiSeq paired-end platform (×300 bp) at Macrogen. Sequencing reads were trimmed based on a quality threshold of 30, and reads shorter than 100 nucleotides were removed using Sickle (34). Good’s coverage for the metagenomic sequencing reads of each sample was calculated using the Nonpareil 3 program (35–38). Other sequencing reads besides bacterial and fungal reads were eliminated using Kaiju (39) against a nonredundant BLAST database. The sequencing reads were assembled using metaSPAdes (v3.12.0) with default parameters (40), and the assembled contigs were grouped into bins to MAGs using MyCC (http://sourceforge.net/projects/sb2nhri/files/MyCC/) with default parameters, based on the nucleotide frequency and read coverage of assembled contigs (41). MAGs were phylogenetically classified using Kaiju, and their qualities were assessed based on lineage-specific marker genes using CheckM (v1.0.4) (42). Only high-quality MAGs with >90% completeness and a <5% contamination rate were used for further analyses.

### Microbe isolation, genome sequencing, and metagenome-based community analysis.

To isolate *Tetragenococcus*, *Chromohalobacter*, *Marinobacter*, Aspergillus, *Debaryomyces*, and *Wickerhamomyces* strains that were dominant in ganjang but for which poor-quality MAGs were produced by the metagenome assembly, ganjang samples were spread onto Trypticase soy agar (BD, USA), marine agar (BD), and MRS agar (BD) media containing 7% (wt/vol) NaCl (for bacteria) and yeast extract-peptone-dextrose agar (BD) medium containing 7% NaCl, chloramphenicol (50 μg/ml), and tetracycline (10 μg/ml) (for fungi), and taxonomically classified through their 16S rRNA or ITS gene sequencing, as described previously (43, 44). The genomes of *Tetragenococcus*, *Chromohalobacter*, *Marinobacter* strains were sequenced using an Illumina HiSeq platform (×150 bp) and Oxford Nanopore MinION sequencer and *de novo* assembled using SOAPdenovo2 and Unicycler (v0.4.7), respectively (45, 46). The genomes of Aspergillus, *Debaryomyces*, and *Wickerhamomyces* strains were sequenced using a PacBio Sequel system (Macrogen) and assembled, according to the procedures described previously (33, 43). The genomes of the isolates, along with high-quality MAGs, were used for further metagenomics and metatranscriptomic analyses (Table 1).

Microbial abundances in the ganjang solution during fermentation were analyzed by mapping the metagenomic sequencing reads to the isolate genomes and high-quality MAGs using the BWA-MEM program based on best-match criteria with a 90% minimum identity and a 20-bp minimum alignment (47). Abundances were normalized based on the genome sizes of the ganjang microbes and are indicated in a relative manner based on the total numbers of mapped reads. The absolute bacterial and fungal abundances were calculated by multiplying the relative bacterial and fungal abundances by the numbers of 16S rRNA or ITS gene copies derived from qPCR analysis and dividing by the numbers of 16S rRNA or ITS gene copies in the genomes.

### Total RNA extraction, sequencing, preprocessing, and metatranscriptomic analysis.

For comparative transcriptional analysis among the samples, a constant amount of spike-in RNA was added to each ganjang pellet sample, and total RNA was extracted using the hot phenol method described previously, with some modifications (48). For the preparation of spike-in RNA, an olfactory receptor gene (*salmOR300-2*, GenBank accession number FJ613852) was PCR amplified from salmon sperm DNA using a primer set, OR300f (5′-CGG GAT CCA TGT CAG CTG GGA ATC-3′) and OR300r (5′-CCG CTC GAG TCA AGA TAC AGC AGG-3′), inserted into a PET28a vector, and transformed into Escherichia coli BL21(DE3) Star. The *salmOR300-2*-cloned PET28a plasmid was extracted from E. coli cells, and the *salmOR300-2* gene in the PET28a plasmid was *in vitro* transcribed using a MEGAscript T7 transcription kit (Invitrogen, USA) according to the manufacturer’s instructions. The purified *salmOR300-2* RNA was used as the spike-in RNA for sample-to-sample normalization.

Aliquots (2 μg) of *salmOR300-2* RNA were spiked into each ganjang pellet (derived from 3 ml of ganjang solution, 1 ml from each ganjang batch) samples resuspended in preheated (65°C) 750 μl TES buffer (10 mM Tris-HCl [pH 7.5], 10 mM EDTA, and 0.5% SDS) and 750 μl phenol-chloroform (5:1, pH 4.7; Sigma-Aldrich). The resulting mixture was homogenized using a FastPrep-24 bead beater (MP Biomedicals, USA) for 60 s at a speed setting of 6.0 and then centrifuged for 10 min (16,100 × *g*, 4°C). The upper aqueous phase was transferred to a fresh microcentrifuge tube and purified as described previously (48). rRNA subtraction from the purified total RNA and cDNA library construction were performed using a TruSeq total RNA sample prep kit (Illumina) according to the manufacturer's instructions, and the resulting cDNA libraries were sequenced using an Illumina HiSeq platform (×101 bp) at Macrogen. The cDNA sequencing reads were trimmed, and those shorter than 30 nucleotides were removed using Sickle. Good’s coverage for the metatranscriptomic sequencing reads of each sample was calculated using Nonpareil 3 program (35–38). Structural RNA (rRNA and tRNA) reads were removed from the clean cDNA reads by BLASTN comparisons with a cutoff criterion of an E value of ≤0.001 against rRNA and tRNA genes, as described previously (48). The transcriptional expression of microbes in the ganjang solution during fermentation was analyzed by mapping the putative mRNA reads to the isolate genomes and high-quality MAGs using the BWA-MEM program, based on best-match criteria with a 90% minimum identity and a 20-bp minimum alignment, and indicated in a relative manner based on the total numbers of mapped reads. The transcriptional expression among the ganjang samples was normalized based on the sequenced read numbers of spike-in RNA in a sample.

Functional genes of the ganjang microbiomes were identified from the isolate genomes and high-quality MAGs using Prokka (v1.12) with default parameters (49), and their predicted protein sequences were submitted to BlastKOALA (http://www.kegg.jp/blastkoala/) (50) for functional annotation. The transcriptional expression of functional genes in each KEGG category during fermentation is indicated as the sum of the read numbers per kilobase of each coding sequence per million mapped reads (RPKM) values of mRNA reads best mapped onto the genes of the isolate genomes and high-quality MAGs assigned to each KEGG functional category. In addition, metabolic pathways of the ganjang microbiomes were generated using the iPath v3 module (https://pathways.embl.de/) (51), based on the KEGG Orthology (KO) numbers of the functional genes, and best mapping of the normalized bacterial and fungal mRNA transcripts against the KEGG metabolic pathways of the isolate genomes and high-quality MAGs was performed. The transcriptional levels of the KEGG pathways of bacteria and fungi during fermentation were depicted relatively using different line thickness and color brightness based on the sum and normalization (based on the sequenced read numbers of spike-in RNA in each sample) of the RPKM values of all functional genes.

### Metabolic pathway reconstructions and metatranscriptomic analyses of the ganjang microbiome.

Carbohydrate, protein, and lipid metabolic pathways were reconstructed based on the predicted KEGG pathways and the Enzyme Commission numbers of the functional genes of the isolate genomes and high-quality MAGs. In addition, the presence or absence of metabolic genes in each genome was manually curated through BLASTP analysis of the reference protein sequences of other closely related strains available in the UniProt database (https://www.uniprot.org) against each genome. The transcriptional expression of each gene related to the various metabolic pathways during fermentation was investigated by mapping bacterial and fungal mRNA transcripts to the isolate genomes and high-quality MAGs using the BWA tool, and their transcriptional expression was visualized using pie charts based on their normalized RPKM values.

### Data availability.

The Illumina sequencing data of the bacterial 16S rRNA genes, fungal ITS genes, metagenomes, and metatranscriptomes derived in this study are publicly available in the NCBI Sequence Read Archive (SRA) under accession numbers SRR11362474 to SRR11362493 and SRR11362474 to SRR11362478 (NCBI BioProject accession number PRJNA613738).
