# Supplementary figures and images for: Metabolic Features of Ganjang (a Korean Traditional Soy Sauce) Fermentation Revealed by Genome-Centered Metatranscriptomics
Source: mSystems. 2021 Aug 3;6(4):e00441-21. doi: 10.1128/mSystems.00441-21 (PMC8407349; doi:10.1128/mSystems.00441-21)

**Supplementary Fig. S1**

**
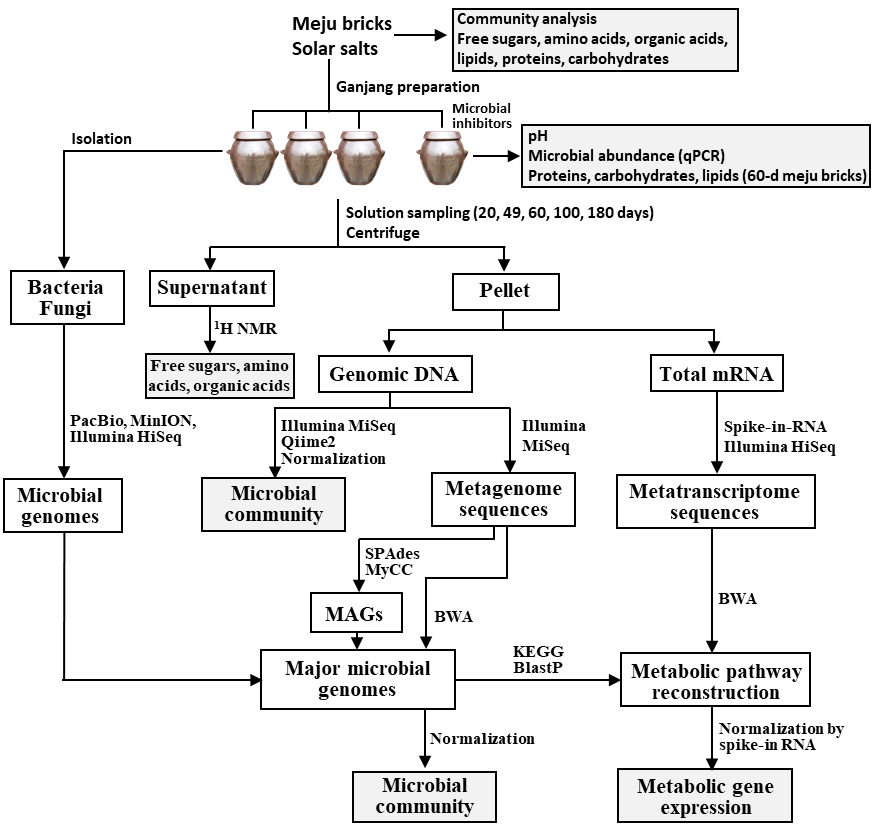
**

Supplement: FIG S1 [file msystems.00441-21-sf001.docx]

**Supplementary Fig. S2**


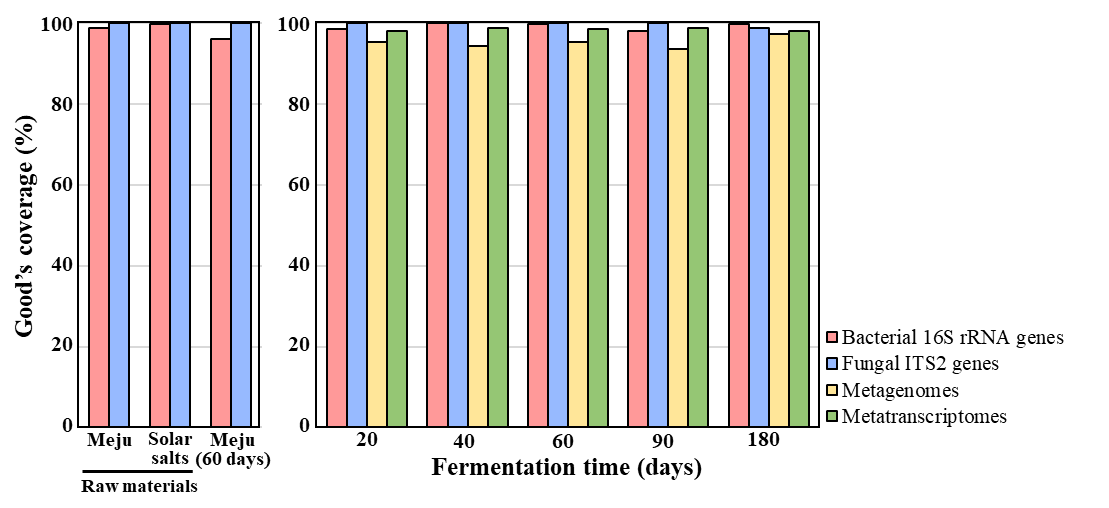

Supplement: FIG S2 [file msystems.00441-21-sf002.docx]

**Supplementary Fig. S3**


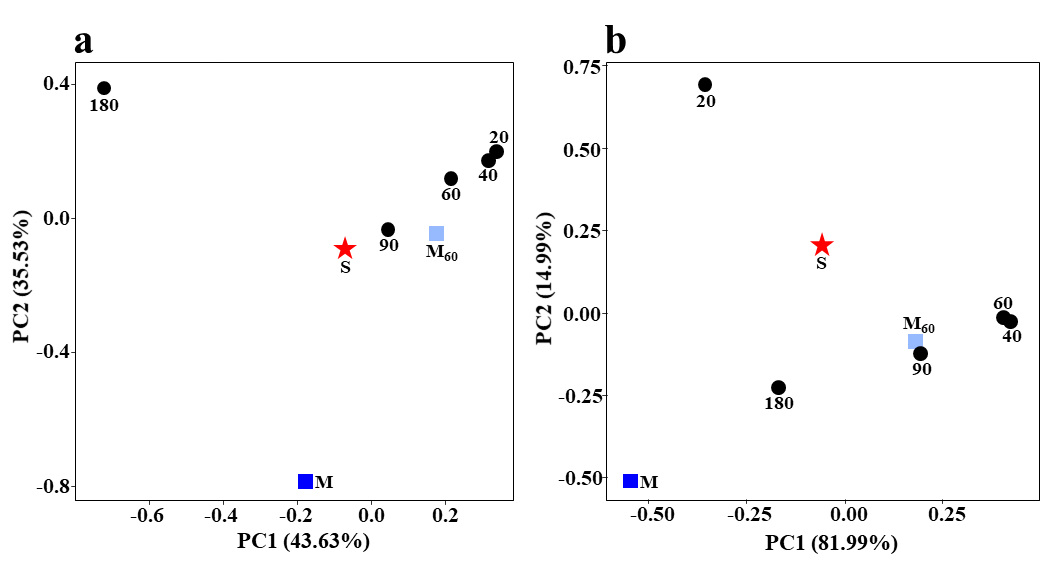

Supplement: FIG S3 [file msystems.00441-21-sf003.docx]

**Supplementary Fig. S4**


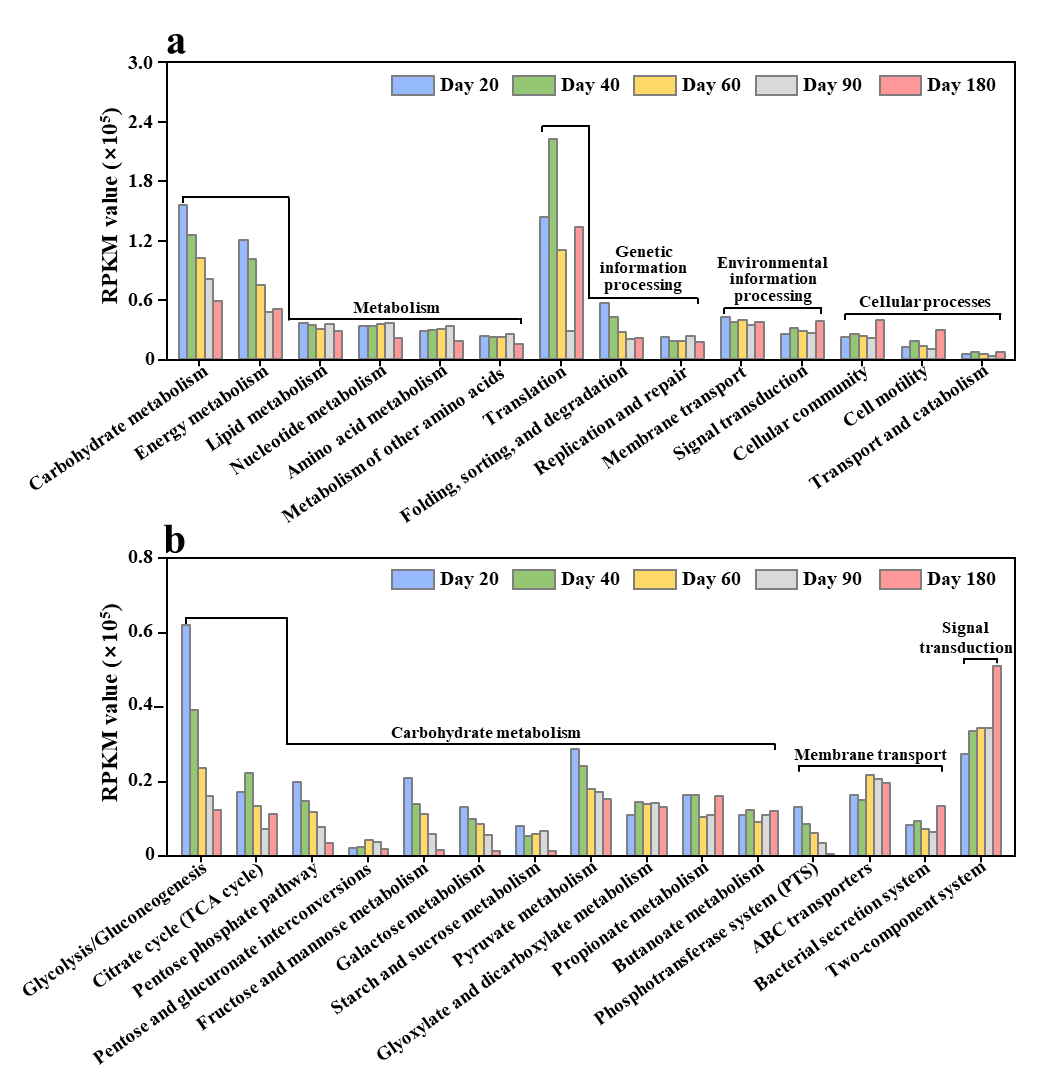

Supplement: FIG S4 [file msystems.00441-21-sf004.docx]

**Supplementary Fig. S5**

**
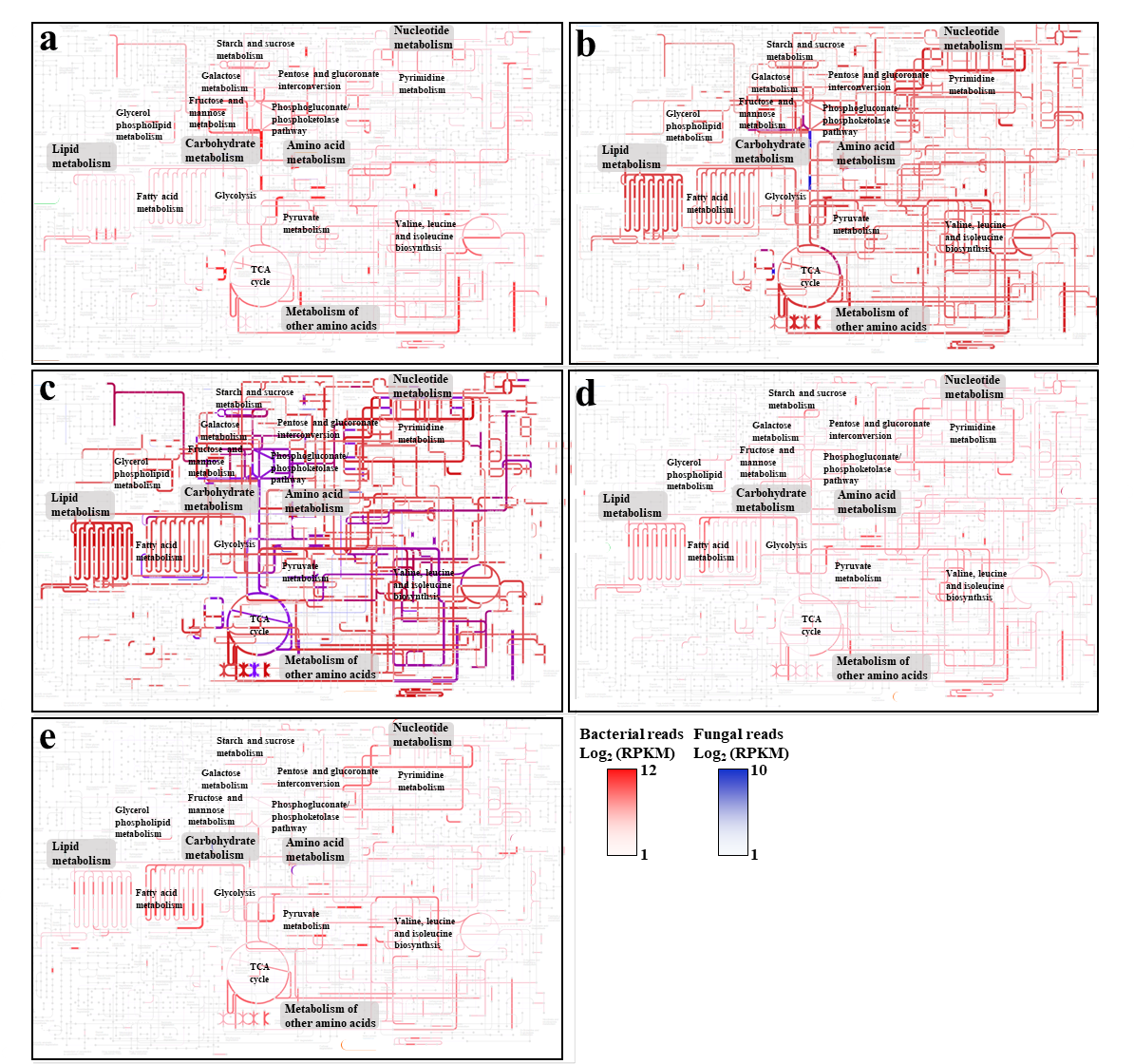
**

Supplement: FIG S5 [file msystems.00441-21-sf005.docx]
