# Supplementary material for: Metabolic Features of Ganjang (a Korean Traditional Soy Sauce) Fermentation Revealed by Genome-Centered Metatranscriptomics
Source: mSystems. 2021 Aug 3;6(4):e00441-21. doi: 10.1128/mSystems.00441-21 (PMC8407349; doi:10.1128/mSystems.00441-21)
Supplement: TABLE S1 [file msystems.00441-21-st001.docx]

**Supplementary Table S1**

| Sample  (days) | *Bacteria* | | | |  | *Fungi* | | | |
| --- | --- | --- | --- | --- | --- | --- | --- | --- | --- |
|  | High- quality reads | OTU**^a^** | Chao1**^a^** | Shannon-Weaver**^a^** |  | High-quality reads | OTU**^a^** | Chao1**^a^** | Shannon-Weaver**^a^** |
| M | 362 | 25 | 27.5 | 4.15 |  | 235,123 | 13 | 14.2 | 0.5 |
| S | 605 | 38 | 39.0 | 4.2 |  | 94,283 | 168 | 171.2 | 4.7 |
| 20 | 4,360 | 70 | 74.3 | 5.0 |  | 4,594 | 17 | 17.0 | 2.5 |
| 40 | 5,152 | 71 | 76.5 | 5.1 |  | 1,990 | 12 | 10.3 | 1.9 |
| 60 | 10,934 | 111 | 121.9 | 5.3 |  | 1,350 | 19 | 19.0 | 3.2 |
| 90 | 5,664 | 122 | 125.9 | 5.8 |  | 16,580 | 31 | 31.3 | 2.2 |
| 180 | 8,403 | 97 | 106.7 | 4.8 |  | 45,232 | 135 | 143.5 | 3.9 |
| M_60_ | 5,466 | 11 | 11 | 1.95 |  | 84,074 | 11 | 12.9 | 1.3 |

OTU, operational taxonomic unit.

**^a^** The statistical diversity indices in each sample were calculated at a 3% distance level using normalized bacterial and fungal sequencing reads of 362 and 1,350, respectively.
