# Supplementary material for: Metabolic Features of Ganjang (a Korean Traditional Soy Sauce) Fermentation Revealed by Genome-Centered Metatranscriptomics
Source: mSystems. 2021 Aug 3;6(4):e00441-21. doi: 10.1128/mSystems.00441-21 (PMC8407349; doi:10.1128/mSystems.00441-21)
Supplement: TABLE S2 [file msystems.00441-21-st002.docx]

**Supplementary Table S2**

| Bin no. | Taxonomic classification^a^ | Size (Mb) | G+C contents (%) | Completeness (%)^b^ | Contamination rate (%)^b^ |
| --- | --- | --- | --- | --- | --- |
| 1 | *Bacillus* | 3.1 | 46.7 | 94.5 | 1.7 |
| 2 | *Bacillus* | 3.1 | 37.6 | 92.8 | 2.8 |
| 3 | *Bacillus* | 4.5 | 45.5 | 98.7 | 4.4 |
| 4 | *Corynebacterium* | 3.0 | 68.9 | 99.8 | 3.9 |
| 5 | *Staphylococcus* | 3.1 | 32.9 | 98.8 | 3.9 |
| 6 | *Halomonas* | 3.0 | 64.7 | 92.6 | 3.7 |
| 7 | *Virgibacillus* | 3.6 | 36.5 | 93.8 | 4.0 |
| 8 | *Alteromonadaceae* | 4.9 | 54.5 | 93.2 | 3.6 |
| 9 | *Idiomarinaceae* | 4.2 | 46.5 | 99.4 | 2.0 |
| 10 | *Micrococcaceae* | 3.1 | 56.5 | 95.0 | 2.5 |
| 11 | *Tetragenococcus* | 2.7 | 35.3 | 90.1 | 17.2 |
| 12 | *Chromohalobacter* | 2.8 | 64.7 | 82.8 | 17.0 |
| 13 | *Marinobacter* | 4.2 | 54.6 | 82.9 | 27.4 |
| 14 | *Debaryomyces* | 8.5 | 36.0 | – | – |
| 15 | *Wickerhamomyces* | 3.1 | 34.4 | – | – |

^a^ MAGs were taxonomically classified using the web-based program Kaiju (<http://kaiju.binf.ku.dk/>).

^b^MAGs 1–10 with >90% completeness and <5% contamination rate were used for further analyses in this study.
