# Supplementary material for: Metabolic Features of Ganjang (a Korean Traditional Soy Sauce) Fermentation Revealed by Genome-Centered Metatranscriptomics
Source: mSystems. 2021 Aug 3;6(4):e00441-21. doi: 10.1128/mSystems.00441-21 (PMC8407349; doi:10.1128/mSystems.00441-21)
Supplement: TABLE S3 [file msystems.00441-21-st003.docx]

**Supplementary Table S3**

| Fermentation time (days) | No. of sequencing reads | No. of high-quality reads^a^ | No. of reads mapped to the genomes (%)^b^ |
| --- | --- | --- | --- |
| 20 | 3,977,867 | 3,602,930 | 3,298,931 (91.6) |
| 40 | 8,406,482 | 7,512,719 | 7,028,413 (93.6) |
| 60 | 5,016,888 | 4,854,132 | 4,638,704 (95.6) |
| 90 | 7,569,265 | 6,616,851 | 6,053,117 (91.5) |
| 180 | 7,117,705 | 6,526,950 | 6,478,347 (99.3) |

^a^The high-quality reads include only high-quality bacterial and fungal sequencing reads.

^b^The mapped reads indicate high-quality sequencing reads mapped to the 17 microbial genomes listed in Table 1 and the percentages represent the percentages of mapped reads that were high-quality sequencing reads.
