# Supplementary material for: Metabolic Features of Ganjang (a Korean Traditional Soy Sauce) Fermentation Revealed by Genome-Centered Metatranscriptomics
Source: mSystems. 2021 Aug 3;6(4):e00441-21. doi: 10.1128/mSystems.00441-21 (PMC8407349; doi:10.1128/mSystems.00441-21)
Supplement: TABLE S4 [file msystems.00441-21-st004.docx]

**Supplementary Table S4**

| Fermentation time (days) | No. of cDNA reads | No. of high-quality cDNA reads^a^ | No. of putative mRNA reads^b^ | No. of mRNA reads mapped to the genomes (%)^c^ |
| --- | --- | --- | --- | --- |
| 20 | 60,272,890 | 54,793,537 | 51,957,088 | 32,964,423 (87.7) |
| 40 | 61,838,213 | 56,216,558 | 54,359,999 | 43,014,410 (88.6) |
| 60 | 36,748,048 | 33,407,317 | 27,401,652 | 19,352,542 (76.9) |
| 90 | 78,455,171 | 71,322,883 | 69,845,613 | 54,758,960 (78.4) |
| 180 | 57,259,720 | 52,054,291 | 51,132,605 | 43,878,104 (88.5) |

^a^The high-quality cDNA reads include only high-quality bacterial and fungal cDNA reads.

^b^The putative mRNA reads indicate high-quality bacterial and fungal cDNA reads after structural tRNA and rRNA sequencing reads were removed.

^c^The mRNA reads indicate only mRNA reads mapped to the CDS (coding sequence) of the 17 microbial genomes listed in Table 1, and the percentages represent the percentages of mapped reads for high quality sequencing mRNA reads.
